# Super-Resolution Reconstruction of Reservoir Core CT Images via GAN: Advancing Energy Extraction Accuracy

**DOI:** 10.3390/s26113429

**Published:** 2026-05-28

**Authors:** Xiaochang Lv, Yanhui Li

**Affiliations:** 1 Department of Computer Engineering, Northeast Petroleum University, Qinhuangdao 066004, China; dreambuf@nepu.edu.cn; 2 Bohai Rim Energy Research Institute, Northeast Petroleum University, Qinhuangdao 066004, China

**Keywords:** core CT images, generative adversarial network, MFAGAN, multi-scale discriminator

## Abstract

**Highlights:**

**What are the main findings?**

- MFAGAN achieves superior performance over GAN-based baselines in LPIPS, SSIM, subjective image quality, and generalization ability, although its PSNR is slightly lower than that of distortion-oriented methods such as EDSR.
- Under in-distribution testing on the DeepRock-SR dataset, MFAGAN demonstrates stable and consistent objective and subjective performance; its out-of-distribution generalization remains unevaluated and is acknowledged as a current limitation.

**What are the implications of the main findings?**

- The proposed method offers a cost-effective solution to enhance the resolution and clarity of 2D reservoir core CT images, serving as an exploratory step toward more accurate feature analysis in oil and gas exploration and development.
- By integrating multi-scale attention mechanisms with a perceptual loss function, MFAGAN effectively distinguishes high-frequency from low-frequency information, generating sharper edge and texture features that help preserve critical morphological details of the pore space.

**Abstract:**

Accurate characterization of reservoir core porosity and morphology from core CT images is essential for oil and gas exploration, and super-resolution offers a cost-effective means to enhance image clarity. To overcome the limitation of existing pixel-level algorithms in distinguishing high- from low-frequency information, we propose the Multi-scale Fusion Attention Mechanisms of Generative Adversarial Networks (MFAGAN). MFAGAN integrates a residual-in-residual fusion attention module to improve throat structure discrimination, a multi-scale discriminator to stabilize training and boost generator performance, and a perceptual loss to sharpen edges and textures. Comparative experiments demonstrate that MFAGAN achieves superior performance over GAN-based baselines in terms of learned perceptual image patch similarity (LPIPS), structural similarity index (SSIM), subjective image quality, and generalization ability. However, its peak signal-to-noise ratio (PSNR) is slightly lower than that of methods such as EDSR. When tested within the distribution of the DeepRock-SR dataset, MFAGAN demonstrated stable and consistent objective and subjective performance. However, its out-of-distribution generalization ability has not yet been evaluated, which is a clear limitation of the current study. In our future work, we will build upon MFAGAN to further explore 3D super-resolution reconstruction techniques for core CT images, with the aim of advancing the practical application of super-resolution methods in oil and gas exploration and development.

## 1. Introduction

The exploration and development of oil and gas resources in reservoir formations require a highly precise understanding of their unique morphological structures. Digital cores constructed from core CT images serve as vital geological data assets for energy companies to understand underground oil and gas formations and mineral characteristics [1,2]. It is the decisive reference data for judging the development effect and recovery rate through the core porosity in the process of oil and gas resources exploration and development [3]. Core CT imaging technology, with its non-destructive testing and visualization capabilities, directly determines the predictive accuracy and optimization potential of oil and gas extraction process models [4]. The meticulousness of core CT pictures is inhibited by the precision of CT scanning equipment, with significant variations in data accuracy and cost. This often necessitates trade-offs between imaging resolution and field-of-view [5]. Physically enhancing core CT image resolution at the millimeter, micrometer, and nanometer scales is costly. Super-resolution techniques offer a low-cost solution to achieve these objectives by providing higher-resolution, more detailed 2D core CT images, thereby laying a foundation for optimizing production performance in oil and gas exploration and development.

Super-resolution (SR) discusses the methods for creating high-resolution (HR) images from lower-resolution (LR) pictures. Super-resolution techniques first appeared in Francia’s research findings [6]. Deep learning-based super-resolution technologies have undergone continuous iterative development. In 2014, Dong constructed SRCNN, the first three-layer end-to-end convolutional neural network [7]. In 2023, Han proposed the MSRAFFN network, comprising shallow feature extraction, multi-scale feature fusion, and deconvolution upsampling, which demonstrated strong performance on the SISR task [8]. Lin proposed the EAPT super-resolution network, applicable to image classification, object detection, and semantic segmentation [9]. In 2024, White evaluated the super-resolution performance of remote sensing images across different satellite hardware configurations and geographic types based on the EDSR metric [10]. Steinfurth applied GANs to two-dimensional and three-dimensional finite-time Lyapunov fields, enabling the full manifestation of flow structures [11]. Zhu proposed the Multi-Scale Model (TMSDNet) for single-view and multi-view 3D reconstruction [12]. In 2025, Wen et al. developed SAT-Net for super-resolution of low-quality retinal fundus images [13].

In parallel, deep learning-based super-resolution methods were increasingly employed on geological and rock core CT images. Roslin et al. employed a 3D U-Net architecture to develop granite–dacite CT datasets [14]. Karimpouli et al. evaluated super-resolved Berea sandstone images across multiple resolutions using SRDUN [15]. Chen et al. proposed SRCycleGAN for micro-CT super-resolution of real rock samples [16], while Li et al. combined BM4D denoising with Laplacian filtering for reservoir rock CT enhancement [17]. Sun et al. developed the DC-SRGAN framework for THMC-related core CT super-resolution [18], and Murugesu et al. extended GAN-based methods to dynamic fluid-saturated geological CT images [19]. Although these studies demonstrate the potential of deep learning-based SR for geoscientific imaging, challenges remain in effectively distinguishing and enhancing optimum frequency features, for instance, pore throats and microfractures, which are critical for accurate physical property extraction.

To eradicate these limitations, this research offers a Multi-scale Fusion Attention-based Generative Adversarial Network (MFAGAN) for super-resolution reconstruction of reservoir core CT pictures. The foremost contributions of this research are summarized below.

(1) A hybrid channel–spatial attention (HCSA) module based on residual-in-residual architecture is designed to enhance high-frequency feature extraction related to pores and fractures.

(2) A multi-scale discriminator incorporating multi-scale convolutions is developed to improve adversarial training stability and reconstruction fidelity.

(3) Generalization tests on diverse reservoir datasets, including sandstone, carbonate, and coal cores, demonstrate superior static and dynamic performance compared with existing super-resolution methods.

## 2. Proposed Methods

### 2.1. Network Architecture

**Figure 1:**
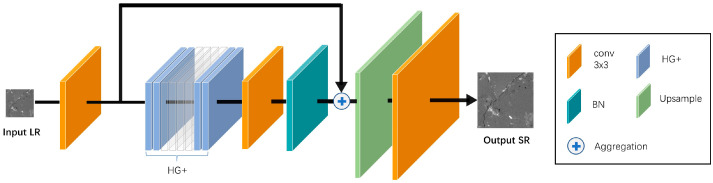
Generator G network structure.

To enhance the ability to extract high- and low-frequency features from CT images of geological cores, this study proposes a Multi-Scale Fusion Attention Generative Adversarial Network (MFAGAN). Experiments confirm MFAGAN’s superior reconstruction quality for 2D core CT images, supporting multi-core super-resolution tasks. The generator architecture appears in Figure 1.

**Figure 2:**
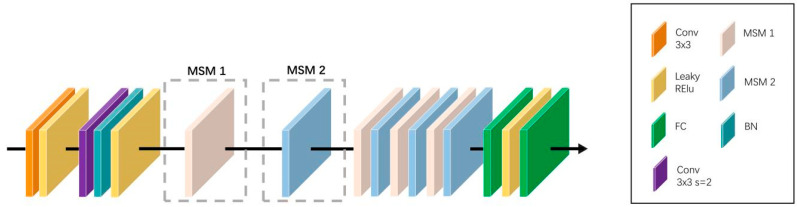
Multi-scale discriminator network architecture.

The discriminator network architecture is shown in Figure 2.

(a) Generator

The generator is made up of shallow feature extraction, deep feature extraction, and image reconstruction (Figure 1).

The shallow feature extraction component performs intricacy operations using a convolutional layer. Convolutional layer parameters are set as follows: 64 convolutional kernels, kernel size of 3 × 3, stride of 1, and padding of 1. Operation process can be expressed as Formula (1).

(1) $$I_{0}=F_{0}(I_{LR})$$

In the equation, $F_{0}$ is convolution action, and $I_{0}$ denotes shallow features, yielding 64 feature maps from conglomerate core CT images. These serve as input to deep feature extraction and the outermost long-skip connection. Padding maintains low-resolution image dimensions via zero-padding at edges.

The module for extracting deep-level features employs a double-layered nested structure. The deep feature extraction component adopts a nested modular design. The outer layer consists of M hybrid groups (HG), each comprising N residual-based HCSA modules and a long residual structure. The i-th input expression for the HG module is defined by Equation (2).

(2) $$G_{i}=I_{i}+F_{1}(H_{i})$$

In the equation, $H_{i}$ denotes output of *i*-th HCSA, $F_{1}$ represents the convolution operation with a 3 × 3 convolution kernel of size 64 and stride 1, yielding 64 feature maps. $I_{i}$ signifies the input to the *i*-th HCSA for implementing residual connections.

Image reconstruction employs sub-pixel convolution for upscaling, utilizing super-resolution magnification factors of ×2 and ×4 for image reconstruction, followed by comparative evaluation of static and dynamic image quality [20].

(b) Discrimator

MFAGAN incorporates an advanced discriminator for robust input evaluation and precise scoring of generator-reconstructed super-resolved images. Balancing generator–discriminator performance is essential in GAN-based super-resolution [21]. An overpowered generator can yield images that saturate discriminator learning, yielding insufficient gradients; conversely, a weak discriminator provides ineffective feedback, hindering generator optimization. Thus, post-generator refinement, the MFAGAN discriminator was enhanced for equilibrium.

The discriminator comprises 8 convolutional modules and 1 fully connected layer (Figure 2). Group 1 applies a 3 × 3 convolution with LeakyReLU activation. Group 2 features a stride-2 3 × 3 convolution for down-sampling, Batch Normalization (BN), and LeakyReLU for nonlinearity. Subsequent groups integrate a fixed-scale multi-scale module (MSM1) and a 1/2 down-sampling multi-scale module (MSM2). Final discrimination uses two fully connected layers. Training follows standard GAN antagonism, optimizing parameters iteratively to elevate core CT reconstruction quality.

### 2.2. Hybrid Channel–Space Attention Module

This research proposes a hybrid residual-based channel and spatial dual attention module (HCSA). It integrates residual convolutions, channel attention, and spatial attention into a fundamental network module for deep feature extraction [22]. The overall hierarchical expression of the module is given by Equation (3).

(3) Hi=Ii+Si(Ci(F0(ReLU(F0(Ii)))))

In the equation, *I_i_* denotes the input from the *i*-th HCSA. After undergoing convolution, ReLU activation, and convolution, it is chronologically fed into *C_i_* (channel attention) and *S_i_* (spatial attention), where the residual sum is computed and output.

**Figure 3:**
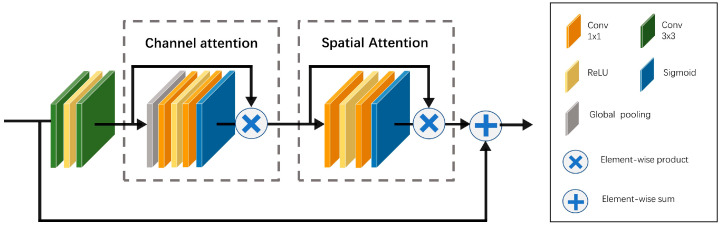
Improved HCSA module structure.

In the HCSA module, the input *I_i_* is processed by two consecutive 3 × 3 convolutions with ReLU activations to extract global features. These features are simultaneously fed into the channel attention module and used as an outer skip connection. The channel attention module employs average pooling, two 1 × 1 convolutions (with ReLU in between), and a Sigmoid function [23]. Its output then enters the spatial attention module, which applies a 3 × 3 convolution, ReLU, a 1 × 1 convolution, and a Sigmoid function to generate spatial weight coefficients. These weights are multiplied element-wise with the original input feature map, and the result is added to the outer skip connection before being output to the next module. The structure of improved HCSA is illustrated in Figure 3.

### 2.3. Multi-Scale Module

The MFAGAN algorithm employs the Inception architecture’s multi-scale convolutions to construct a multi-scale discriminator subnetwork, enhancing the discriminator’s accuracy to achieve balanced synchronous training with the generator subnetwork [24]. First, the MSM1 module was designed for multi-scale feature fusion enhancement, performing multi-scale feature extraction on the SR images reconstructed by the generator. Second, the MSM2 module is designed for multi-scale feature pooling enhancement during the 1/2 downsampling process.

**Figure 4:**
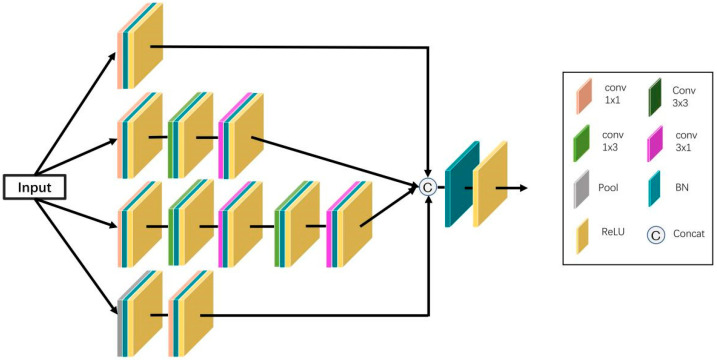
MSM1 module.

In the discriminator architecture, MSM1 is a multi-scale convolution module that maintains the same input-to-output dimensions. The MSM1 module structure is shown in Figure 4.

The MSM1 structure comprises four multi-scale branch structures, each independently deriving feature matrices of identical dimensions and scales. At the output end, these four output matrices are concatenated along depth measurement to generate the output feature map. This map then undergoes activation through a BN layer and LeakyReLU function before being passed to the MSM2 module. The mathematical expression for the MSM1 module is given by Equation (4).

(4) $$Q_{C}=F_{conv}(cat([(F_{1}(I_{x})),(F_{1}(F_{1\times 3}(F_{3\times 1}(I_{x})))),(F_{1}(F_{1\times 3}2(F_{3\times 1}(I_{x})))),(F_{1}(P_{\max}(I_{x})))]))$$

In the equation, $I_{x}$ denotes the input to the MSM1 module, $F_{1}$ represents a convolution operation with a 1 × 1 kernel. $F_{1\times 3}$ and $F_{3\times 1}$ denote convolution operations using 1 × 3 and 3 × 1 heterogeneous convolution kernels, respectively, which are used to replace large convolution kernels and reduce the number of parameters. $P_{\max}$ denotes max pooling operations. cat denotes channel concatenation across the four branches to produce an output with the same number of channels as $I_{x}$. $F_{\mathrm{conv}}$ represents the convolution and activation operations applied to the multi-scale concatenated input, while $Q_{C}$ denotes the output of MSM1.

The four branches initially pass via a 1 × 1 convolution layer, trailed by feature extraction using different types and dimensions of convolution kernels (1 × 1, 3 × 3, 5 × 5, 3 × 3 Pool) to capture structural features of the input feature map across varying receptive fields. High-dimensional feature maps yield local features that converge more readily, while low-dimensional pooling preserves adjacency information while reducing dimensionality to accelerate learning. The construction of large receptive fields typically relies on large-sized convolutional kernels such as 5 × 5 or 7 × 7. However, such kernels lead to an explosive increase in model parameters. Stacking multiple sets of small convolutional kernels serves as an effective alternative to large kernels. In the MSM1 module, this study employs a combination of two sets of 1 × 3 and 3 × 1 convolutional kernels to replace the traditional 5 × 5 kernel. This design effectively diminishes the parameter numbers while sustaining the receptive field size. It also avoids expression bottlenecks and enhances the model’s nonlinear expressive capability. Conceptually, stacking one set of 1 × 3 and 3 × 1 convolutional kernels can homogeneously substitute a single 3 × 3 convolutional kernel; similarly, stacking two sets of 3 × 3 convolutional kernels can homogeneously substitute a single 5 × 5 convolutional kernel.

The parameter comparison for decomposing a single-layer 5 × 5 convolution kernel into two sets of 1 × 3 and 3 × 1 asymmetric convolutions, as shown in Equation (5).

(5) $$\begin{array}{l} Q_{5\times 5}=5\times 5\times C\times C=25C^{2}\\ Q_{5\times 3}=2Q_{3\times 3}=2(3\times 3\times C\times C)=18C^{2}\\ Q_{5\times 3}=4Q_{1\times 3+3\times 1}=2((1\times 3+3\times 1)C\times C)=12C^{2} \end{array}$$

In the formula, C represents the feature map scale. Under identical input and output feature matrix depths, the number of parameters after decomposition is reduced by over 50%. This also lowers computational complexity, particularly in deep networks, enabling lightweight models and highly efficient operation.

**Figure 5:**
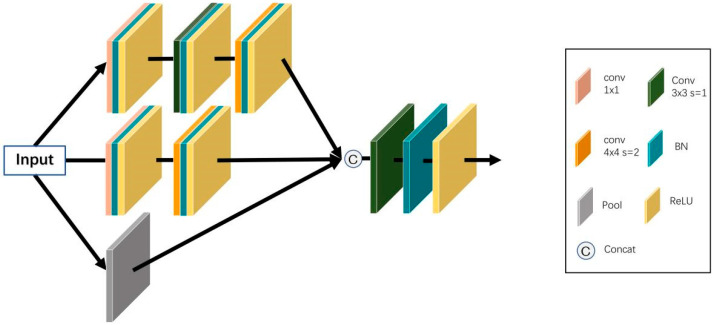
MSM2 module structure.

MSM2 represents a multi-scale convolution module with 1/2 downsampling. The MSM2 module structure is depicted in Figure 5.

MSM2 structure comprises three multi-scale branches. Each branch independently produces two 1/2 down-sampled feature matrices and one 3 × 3 max-pooled feature map. At the output end, these three outputs are concatenated on depth measurement to create a down-sampled multi-channel feature map. Finally, features undergo a 1/2 down-sampling convolutional layer and a BN layer, followed by activation through the LeakyReLU function, before being passed to the MSM2 module. Except for the pooling branch, the other two branches first undergo a 1 × 1 convolutional operation. Subsequently, the features are downsampled by a factor of 1/2 by setting the convolutional stride to 2. The difference between the two branches lies in the inclusion of an additional 3 × 3 full-size convolution before down-sampling in one branch. This enhances feature diversity, enabling more detailed information to be propagated to the merged output layer. This design augments feature diversity, allowing richer fine-grained details to be transmitted to the merged output layer. After obtaining the multi-scale down-sampled merged output, it undergoes a 3 × 3 channel-reduction convolution layer. This is because when feature maps pass through the multi-scale module, the merged channels require reduction to decrease computational load. Subsequently, the 1/2-downsized feature map is output to the next module after passing through a BN layer and LeakyReLU activation.

## 3. Experimental Results

### 3.1. Core CT Image Dataset

(a) CT scan of conglomerate core

The conglomerate samples used in this study came from the Tongbomei Formation in the Wulusun Depression of the Hailar Basin. We used a micro-CT scanner (GE Phoenix Nanotom S) to capture the 3D structure of a 2-mm-diameter conglomerate sample. The scan was run at a tube voltage of 110 kV and a tube current of 100 μA. After reconstruction, we obtained a 3D volume dataset with a resolution of 1700 × 1700 × 1271 voxels. Based on the field of view (FOV) matching the sample size and the image resolution, the voxel size was calculated to be about 1.18 μm (1.176 μm to be precise), and the corresponding spatial resolution was roughly 2.4 μm (or better than 2.5 μm by a conservative estimate).

The 2D CT image slices of the conglomerate core exhibit numerous white areas with high brightness and dense mineral content. The conglomerate core sample contains a diverse array of minerals with well-defined boundaries, revealing a complex structural pattern characterized by matrix, gravel, clay particles, and fractures.

(b) Training Set and Validation Set

The MFAdata dataset was constructed from the conglomerate 2D core CT images. The training, validation, and test sets were split in a ratio of 7:1.5:1.5. First, 1271 original CT images were cropped to 800 × 800 pixels. To prevent data leakage, an interval selection method was applied. The first 800 cropped images were used for training, segmented into 200 × 200 patches, resulting in 12,800 HR sub-images. After skipping 60 images, the next 170 cropped images formed the validation set, yielding 2720 HR sub-images. After skipping another 71 images, the final 170 cropped images formed the test set, also yielding 2720 HR sub-images. LR images in each set were generated by bicubic downsampling to simulate real-world degradation.

### 3.2. Training Details and Parameters

Training used a batch size of 16 for ×4 upscaling. The generator contained 10 HG modules, each with 16 HCSA units. All convolutional layers used a stride of 1 except downsampling layers, with a standard 3 × 3 kernel (padded to preserve dimensions). Shallow feature extraction and HCSA layers used 64 filters/channels. Channel compression factor r = 16, spatial expansion factor i = 2, and upsampling used sub-pixel convolutions. The generator was pre-trained with L1 loss. The learning rate started at 1 × 10^−4^ and was halved at iterations 50 k, 100 k, 200 k, 300 k. The Adam optimizer (β_1_ = 0.9, β_2_ = 0.99) alternately updated the generator and discriminator. The loss function followed ESRGAN [25] with λ = 5 × 10^−3^ and η = 1 × 10^−2^. Reconstruction quality on the MFAdata test set was evaluated using PSNR [26], SSIM [27], and LPIPS [28], comparing MFAGAN with other benchmarks.

### 3.3. Ablation Experiment

This paper conducts comparative experiments on the processing order of the channel attention and spatial attention modules in the HCSA module of MFAGAN. Table 1 summarizes the quantitative results for LPIPS, PSNR, and SSIM on the test set for models selected based on the optimal LPIPS metric after multiple training runs under different processing orders. The results show that executing the channel attention module first and the spatial attention module second reduces the LPIPS value by 0.2922, increases PSNR by 0.6842 dB, while the SSIM metric improves by only 0.0011.

To evaluate the contributions of the MSM1 and MSM2 modules, this paper conducted ablation experiments using a generator network based on ESRGAN and integrated with the HCSA module as the baseline. Based on the model that achieved the optimal LPIPS score across multiple trials, PSNR, SSIM, and LPIPS metrics were obtained on the test set for different module configurations (Table 2). The results indicate that adding MSM1 alone yields a slightly better improvement in LPIPS than adding MSM2 alone, while adding both simultaneously achieves the optimal LPIPS, demonstrating a complementary enhancement effect.

**Figure 6:**
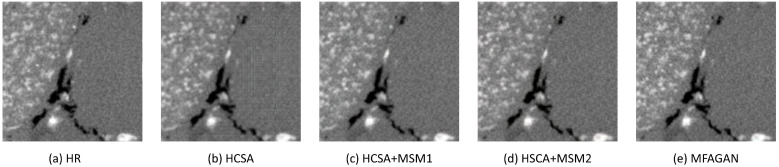
Visual Comparison Results of Different Module Configurations in Ablation Experiments.

Figure 6 presents the results of a visual comparison of different module configurations in ablation experiments, using randomly selected images from the test set; the image reconstruction results exhibit some sample-dependent variability. Among these, the model containing only the HCSA module (b) performs reasonably well on the LPIPS metric, but the reconstructed images contain fairly noticeable artifacts; the visual differences between the HCSA + MSM1 (c) and HCSA + MSM2 (d) variants are minimal; meanwhile, the complete MFAGAN model (e), which includes all modules, exhibits the highest consistency with the ground truth and the best overall visualization results.

### 3.4. Super-Resolution Results of Self-Generated Core CT Images

(a) Objective Evaluation and Analysis

Table 3 summarizes the objective evaluation metrics (PSNR, SSIM, LPIPS) obtained on the test set for each method, where the models were selected based on the optimal LPIPS across multiple training runs. In the ×4 super-resolution reconstruction task, the proposed MFAGAN achieves the lowest LPIPS value (4.2537) among generative adversarial network (GAN)-based methods, indicating its best perceptual quality. In terms of PSNR and SSIM, MFAGAN (28.6466 dB, 0.6042) outperforms SRGAN (28.5635 dB, 0.5914) but is slightly inferior to ESRGAN (28.9967 dB, 0.6445). As a representative distortion-oriented method, EDSR achieves the highest PSNR and SSIM (31.6945 dB, 0.7593) but also the highest LPIPS (7.8204), illustrating the typical trade-off between perceptual quality and distortion metrics.

**Figure 7:**
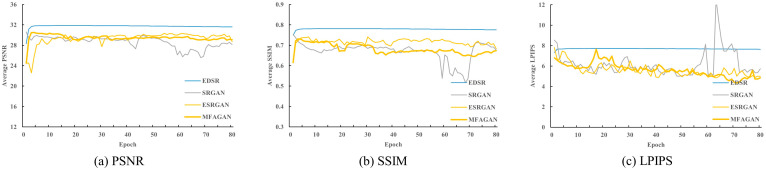
Average PSNR, SSIM, and LPIPS curves for different methods at ×4 magnification.

Figure 7 presents the training curves of PSNR, SSIM, and LPIPS for EDSR, SRGAN, ESRGAN, and the proposed MFAGAN on the MFAdata dataset. As shown in Figure 7a,b, all methods converge quickly and then remain relatively stable. EDSR consistently achieves the highest PSNR and SSIM. MFAGAN and ESRGAN perform similarly, with close and moderately fluctuating curves. SRGAN exhibits a late-stage SSIM drop and a sharp LPIPS spike, indicating training instability. In terms of perceptual quality (Figure 7c), EDSR maintains a consistently high LPIPS. MFAGAN and ESRGAN show gradual LPIPS reduction over epochs. SRGAN’s LPIPS fluctuates significantly, including a large mid-training outlier, suggesting inconsistent artifacts. Overall, MFAGAN achieves competitive and stable performance in both objective and perceptual metrics, demonstrating its effectiveness for 2D super-resolution reconstruction of conglomerate core CT images.

### 3.5. Generalization Ability Testing Results

This section conducts generalization capability tests on the MFAGAN algorithm. In the experiments, all algorithm parameters are retained at their initial settings to ensure the representativeness of the test results. The training epochs are set to 30, with the iteration count set to 42,000.

(a) Dataset and Preprocessing

Sandstone, carbonate, and coal cores are the core targets in oil and gas exploration and development. Their microscopic pore-throat and fracture characteristics directly determine reservoir properties and production capacity, making high-resolution CT imaging essential for precise characterization. Therefore, this section’s generalization capability experiment utilizes sandstone, carbonate, and coal core CT images from the DeepRock-SR dataset, with 4000 images per type [29]. After randomly mixing the 3 core CT image types, 11,200 images were carefully chosen for the exercise set and 800 for the validation set. During training, the core CT images underwent mean-subtracted normalization. Data augmentation was applied to paired HR and LR core CT images by randomly rotating them by fixed angles and horizontally/vertically flipping them. The overall model training curve remained stable. Significant differences in feature representation were observed among carbonate, sandstone, and coal core CT images. Notably, coal images contained a higher proportion of low-frequency information, and some images exhibited low contrast between high- and low-frequency details. However, it should be noted that the generalization assessment in this section is currently limited to in-distribution testing on the DeepRock-SR dataset. Out-of-distribution generalization (e.g., to different rock types, scanning resolutions, or geological formations) has not been evaluated and remains a key direction for future research.

(b) Static test results

Table 4 presents the quantitative evaluation results of different methods at ×4 magnification on the mixed dataset (carbonate, sandstone, and coal cores). Among the GAN-based methods, MFAGAN achieves the highest PSNR (29.6858 dB) and SSIM (0.6384), along with the lowest LPIPS (4.3751), indicating a favorable trade-off between distortion metrics and perceptual quality. ESRGAN ranks second in LPIPS (4.4956) but slightly lower in PSNR/SSIM. SRGAN shows the weakest performance among GAN methods across all three metrics. As a distortion-oriented method, EDSR obtains the highest PSNR (30.2418 dB) and SSIM (0.7068) but also the highest LPIPS (7.8072).

Table 5 presents the parameter counts for the MFAGAN algorithms alongside SRGAN, ESRGAN, EDSR, and other algorithms in the ×4 upscaling task. Separate calculations were performed for the generator and discriminator of the three GAN-based models.

## 4. Subjective Evaluation and Analysis

### 4.1. Discussion on Subjective Evaluation Based on the MFAdata Dataset

**Figure 8:**
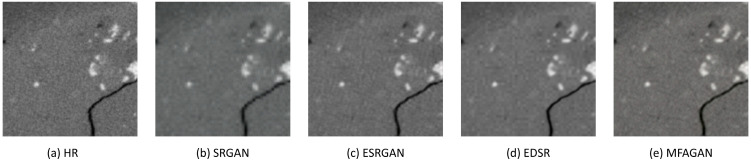
CT image reconstruction of conglomerate cores with different methods at ×4 magnification.

As shown in Figure 8, the MFAGAN algorithm proposed in this paper, along with SRGAN, ESRGAN, and EDSR, demonstrates the differences and enhancement effects between reconstructed SR pictures and original high-resolution HR pictures at ×4 magnification from a subjective visual perspective.

In Figure 8, the (a) original HR image derived from the conglomerate core CT scan reveals that high-frequency features such as fractures and minerals constitute a small proportion overall. A slight crack is visible, and the mineral-related bright spots show varying degrees of brightness. Among the super-resolution reconstructed images produced by different methods, MFAGAN demonstrates high subjective visual fidelity in restoring fractures and spots, with no loss of fine fracture details. The SRGAN algorithm exhibits blurring and blocky artifacts, resulting in poor reconstruction quality. The end-to-end network EDSR achieves relatively clear reconstruction of larger fractures, but its smoothing effect leads to loss of fine fracture details.

### 4.2. Discussion on Subjective Evaluation Based on the DeepRock-SR Dataset

**Figure 9:**
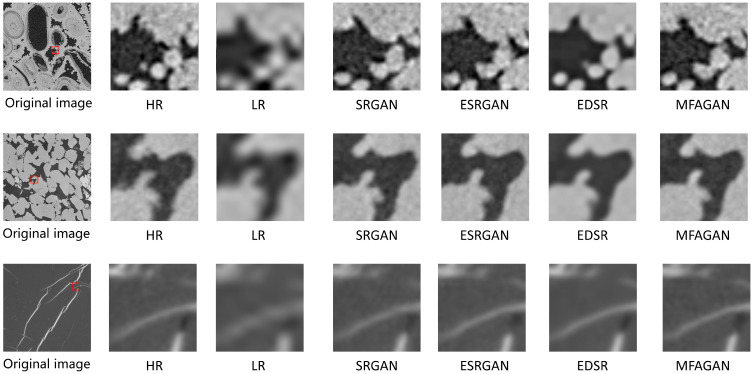
Comparison of super-resolved core CT images reconstructed by different methods. The location of the HR image in the original image is indicated by the red box.

After reconstructing CT images of carbonate, sandstone, and coal cores from the validation set using different algorithms, selected reconstructed CT images were extracted for visual classification comparison, as shown in Figure 9. Although the MFAGAN algorithm designed in this paper yields lower PSNR and SSIM objective evaluation metrics than the end-to-end EDSR algorithm, subjective comparisons of reconstructed images reveal that MFAGAN produces superior visual quality compared to the other four methods. The reconstructed images from the four perception loss-based algorithms MFAGAN, SRGAN, and ESRGAN closely resemble the original HR images. In contrast, the EDSR reconstructed images exhibit noticeable differences from the original HR images, appearing smoother with missing fine details.

All three types of core CT super-resolution images contain substantial high-frequency information, including fractures, mineral distributions, and edge textures. Significant differences exist among the algorithms in reconstructing fine fractures: the SRGAN algorithm exhibits feature loss in reconstructed images; the EDSR algorithm produces reconstructed images with smoothed edge textures; the MFAGAN algorithm yields reconstructed images with clearer high-frequency feature edges.

Figure 9 shows a detailed magnification comparison of the ×4 magnification super-resolution reconstructed core CT image. This figure more clearly demonstrates the detailed reconstruction and restoration effects of various algorithms. Across different core CT image reconstruction tasks, the MFAGAN algorithm exhibits feature contrast and detail restoration levels that more closely resemble the original high-resolution (HR) core CT images.

## 5. Conclusions

This paper proposes a 2D Multi-Scale Generative Adversarial Network (MFAGAN) for super-resolution reconstruction of real geological core CT images. It is applied to reconstruct 2D super-resolution images of conglomerate core CT data from the Hailar Basin, providing technical support for oil and gas exploration and development. First, an HCSA module is designed based on residual-in-residual hybrid channel attention and spatial attention mechanisms to enhance the generator’s SR image reconstruction capability. Second, multi-scale MSM1 and MSM2 modules are designed to enhance the discriminator’s ability to distinguish input SR images, enabling balanced synchronous training of the generative adversarial network. Sub-pixel convolutions achieve super-resolution reconstruction of conglomerate core CT images at a magnification level of ×4. The generalization capability of MFAGAN is tested on the DeepRock-SR dataset, yielding competitive objective and subjective evaluations under the in-distribution setting. In future work, we will further investigate 3D super-resolution techniques, along with their computational complexity and inference costs, to enable the practical application of super-resolution technology in real exploration and development.

## Figures and Tables

**Table 1:** Test results for different sequences of the Channel Attention Module (CA) and Spatial Attention Module (SA) in the HCSA module at ×4 magnification.

	Magnification	PSNR	SSIM	LPIPS
CA + SA	×4	28.6466	0.6042	4.2537
SA + CA	×4	27.9624	0.6031	4.5469

**Table 2:** Ablation results of different modules. √ indicates that the corresponding module is being used.

HCSA	MSM1	MSM2	PSNR	SSIM	LPIPS
√			27.2485	0.5704	4.4315
√	√		28.3248	0.6173	4.4018
√		√	28.4613	0.6194	4.5385
√	√	√	28.6466	0.6042	4.2537

**Table 3:** Relative outcomes of diverse approaches applied to MFAdata dataset of CT images of conglomerate cores.

Method	Magnification	PSNR (dB)	SSIM	LPIPS
EDSR	×4	31.6945	0.7593	7.8204
SRGAN	×4	28.5635	0.5914	5.0698
ESRGAN	×4	28.9967	0.6445	4.6296
MFAGAN	×4	28.6466	0.6042	4.2537

**Table 4:** Comparison results of 4 methods at different magnifications.

Method	Magnification	PSNR (dB)	SSIM	LPIPS
EDSR	×4	30.2418	0.7068	7.8072
SRGAN	×4	28.3593	0.6158	5.3205
ESRGAN	×4	29.3482	0.6375	4.4956
MFAGAN	×4	29.6858	0.6384	4.3751

**Table 5:** Number of parameters for 5 methods at different multiplicities.

Method	SRGAN	ESRGAN	MFARGAN	EDSR
Magnification	×4	×4	×4	×4
Generator (M)	1.52	16.70	36.79	43.02
Discriminator (M)	15.50	14.50	32.16	~

## Data Availability

The datasets used and analyzed during the current study are available from the corresponding author on reasonable request.

## Abbreviations

The following abbreviations are used in this manuscript:

BM4D	Block-Matching and 4D Filtering
BN	Batch Normalization
DC-SRGAN	Deep Convolutional Super-Resolution Generative Adversarial Network
EAPT	Efficient Attention Pyramid Transformer
EDSR	Enhanced Deep Super-Resolution Network
ESRGAN	Enhanced Super-Resolution Generative Adversarial Networks
FAGAN	Fusion Attention of Generative Adversarial Networks
GAN	Generative Adversarial Network
HCSA	Hybrid Channel-Spatial Attention
HR	High-Resolution
LR	Low-Resolution
MAE	Mean Absolute Error
MFAGAN	Multi-Scale Fusion Attention Mechanisms of Generative Adversarial Networks
MSE	Mean Squared Error
MSM1	Fixed-Scale Multi-Scale Module
MSM2	1/2 Downsampling Multi-Scale Module
MSRAFFN	Multi-Scale Recursive Attention Feature Fusion Network
PSNR	Peak Signal-to-Noise Ratio
SAT-Net	Structure-Aware Transformer-Based Attention Fusion Network
SR	Super-Resolution
SRCNN	Super-Resolution Convolutional Neural Network
SRCycleGAN	Super-Resolution Cycle-Consistent Generative Adversarial Network
SRDUN	Super-Resolution Dense U-Net
SRGAN	Super-Resolution Generative Adversarial Network
SSIM	Structural Similarity Index
TMSDNet	Transformer with Multi-Scale Dense Network
U-Net	U-Net Convolutional Neural Network
VGG	Visual Geometry Group Network
